# Ancient Views Concerning Earthquakes

**Published:** 1882-06

**Authors:** 


### ANCIENT VIEWS CONCERNING EARTHQUAKES.

Anaxagoras, the Rhodian, held that *earthquakes* are nothing but a sort of cosmic flatulence—winds which have strayed into caverns, where they cannot find an outlet. Aristotle ascribes them to vapors generated by the infiltration of water through the fissures of a rocky sea-bottom ; and Pliny, to the pressure of air confined in deep caves, and reacting against the collapse of superincumbent rock-strata. But the most ingenious explanation is offered by St. Thomas of Aquinas. Earthquakes, he suggests, may be caused by the struggles of defunct misbelievers, trying (by a simultaneous stampede, perhaps) to escape from the pit of torment.—*Popular Science Monthly.*

